# Dexamethasone sensitizes to ferroptosis by glucocorticoid receptor–induced dipeptidase-1 expression and glutathione depletion

**DOI:** 10.1126/sciadv.abl8920

**Published:** 2022-02-02

**Authors:** Anne von Mässenhausen, Nadia Zamora Gonzalez, Francesca Maremonti, Alexia Belavgeni, Wulf Tonnus, Claudia Meyer, Kristina Beer, Monica T. Hannani, Arthur Lau, Mirko Peitzsch, Paul Hoppenz, Sophie Locke, Triantafyllos Chavakis, Rafael Kramann, Daniel A. Muruve, Christian Hugo, Stefan R. Bornstein, Andreas Linkermann

**Affiliations:** 1 Division of Nephrology, Department of Internal Medicine 3, University Hospital Carl Gustav Carus at the Technische Universität Dresden, Dresden, Germany.; 2 Biotechnology Center, Technische Universität Dresden, 01307 Dresden, Germany.; 3 Clinic for Renal and Hypertensive Disorders, Rheumatological and Immunological Disease, University Hospital of the RWTH Aachen, Aachen 52074, Germany.; 4 Heidelberg University, Faculty of Medicine, and Heidelberg University Hospital, Institute for Computational Biomedicine, Bioquant, Heidelberg, Germany.; 5 Department of Medicine, University of Calgary, Calgary, Canada.; 6 Snyder Institute for Chronic Disease, University of Calgary, Calgary, Canada.; 7 Institute of Clinical Chemistry and Laboratory Medicine, University Hospital Carl Gustav Carus at the Technische Universität Dresden, Fetscherstrasse 74, Dresden 01307, Germany.; 8 Department of Internal Medicine, Nephrology and Transplantation, Erasmus Medical Center, 3015 GD Rotterdam, Netherlands.; 9 Department of Internal Medicine 3, University Hospital Carl Gustav Carus at the Technische Universität Dresden, Dresden, Germany.; 10 Diabetes and Nutritional Sciences, King’s College London, London, UK.; 11 Center for Regenerative Therapies, Technische Universität Dresden, Dresden, Germany.; 12 Paul Langerhans Institute Dresden of Helmholtz Centre Munich at University Clinic Carl Gustav Carus of TU Dresden Faculty of Medicine, Dresden, Germany.; 13 Lee Kong Chian School of Medicine, Nanyang Technological University, Singapore, Singapore.

## Abstract

Dexamethasone is widely used as an immunosuppressive therapy and recently as COVID-19 treatment. Here, we demonstrate that dexamethasone sensitizes to ferroptosis, a form of iron-catalyzed necrosis, previously suggested to contribute to diseases such as acute kidney injury, myocardial infarction, and stroke, all of which are triggered by glutathione (GSH) depletion. GSH levels were significantly decreased by dexamethasone. Mechanistically, we identified that dexamethasone up-regulated the GSH metabolism regulating protein dipeptidase-1 (DPEP1) in a glucocorticoid receptor (GR)–dependent manner. DPEP1 knockdown reversed the phenotype of dexamethasone-induced ferroptosis sensitization. Ferroptosis inhibitors, the DPEP1 inhibitor cilastatin, or genetic *DPEP1* inactivation reversed the dexamethasone-induced increase in tubular necrosis in freshly isolated renal tubules. Our data indicate that dexamethasone sensitizes to ferroptosis by a GR-mediated increase in DPEP1 expression and GSH depletion. Together, we identified a previously unknown mechanism of glucocorticoid-mediated sensitization to ferroptosis bearing clinical and therapeutic implications.

## Teaser

Dexamethasone leads to GR-mediated increased DPEP1 expression and GSH depletion, resulting in higher ferroptosis sensitivity.

## INTRODUCTION

Glucocorticoids are used in clinical routine to treat leukemias (*1*, *2*) and COVID-19 (*3*), for immunosuppression during solid organ transplantation (*4*), and in conjunction with cancer radiation therapy or, e.g., cataract surgery to reduce edema in critical areas (*5*). Dexamethasone exhibits a favorably 25-fold higher anti-inflammatory potency compared with cortisol (*6*), but the use of dexamethasone is limited by several dose-dependent side effects, including but not limited to osteoporosis and osteonecrosis (*7*), myopathy (*8*), peptic ulcers (*9*), and growth retardation (*10*).

We and others (*11*, *12*) recently reported exquisite sensitivity of steroid-producing adrenocortical carcinomas to ferroptosis induction. Ferroptosis was first reported to be induced by erastin, an inhibitor of system Xc^−^, a plasma membrane antiporter involved in the regulation of glutathione (GSH) concentrations (*13*). Ferroptosis is an iron-catalyzed form of regulated necrosis (*13*, *14*) that is critically involved in the pathophysiology of myocardial infarction (*15*–*17*), acute stroke (*18*–*20*), neurodegeneration (*21*), kidney injury (*22*–*24*), and in the context of transplantation (*25*–*27*).

Given the potential role of necrotic cell death in untoward effects of steroids, we hypothesized that dexamethasone might control signaling pathways of regulated necrosis. We found dexamethasone and other glucocorticoids to lower the threshold to ferroptosis by decreasing GSH content in a glucocorticoid receptor (GR)–dependent manner. By means of RNA sequencing (RNA-seq), we identified the glutathione regulator dipeptidase-1 (DPEP1) to mediate the ferroptosis-sensitizing effect by dexamethasone.

## RESULTS

### Dexamethasone sensitizes to erastin-induced ferroptosis but not to RSL3-induced ferroptosis

**Fig. 1.**
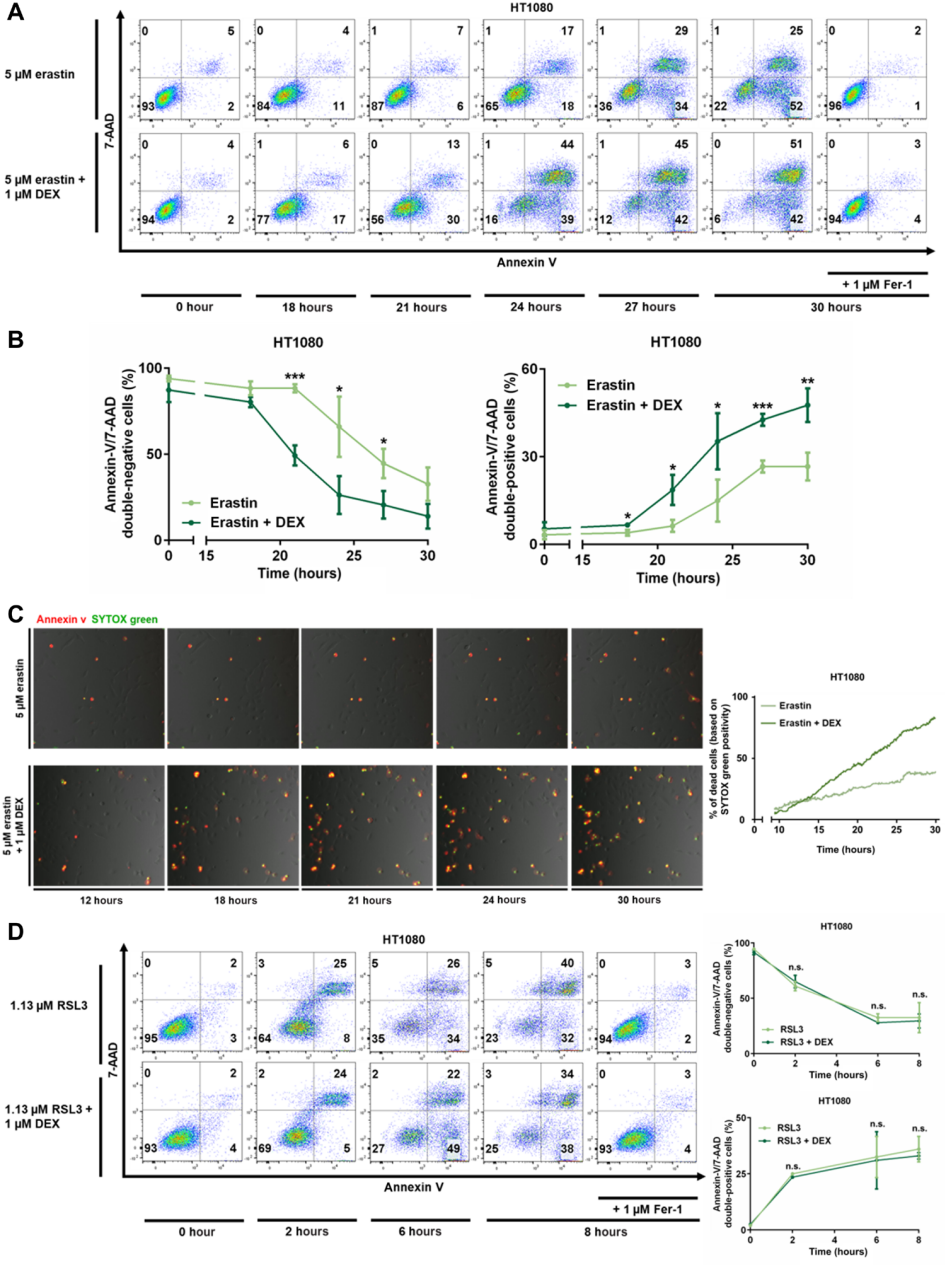
Dexamethasone sensitizes to erastin-induced, but not to RSL3-induced, ferroptosis. (**A**) HT1080 cells were treated for indicated times with 5 μM erastin and 1 μM Fer-1 with or without pretreatment of 1 μM dexamethasone for 12 hours. 7-AAD and annexin V were read out by fluorescence-activated cell sorting (FACS). (**B**) Quantification of data presented in (A) and repetitions of this experiment. (**C**) Still images of indicated times of a double-chamber time-lapse imaging and quantification of SYTOX-positive cells over time in HT1080 cells pretreated with dexamethasone as indicated. (**D**) HT1080 cells were induced to undergo ferroptosis by the GSH peroxidase 4 (GPX4) inhibitor RSL3 and assessed as in (A) after indicated times. Experiments were repeated at least three times, and representative images and FACS plots are shown. The graphs show means ± SD. Statistical analysis was performed using Student’s *t* test for each time point. *P ≤ 0.05, **P ≤ 0.01, ***P ≤ 0.001, n.s., nonsignificant.

Dexamethasone was previously demonstrated to induce intrinsic apoptosis in selected cells, such as primary murine thymocytes and RS4;11 cells. In our hands, dexamethasone caused fractions of thymocytes to become annexin V positive, and this effect was reversed by the caspase inhibitor emricasan (fig. S1A). Similarly, RS4;11 cells externalized phosphatidylserine to the outer leaflet of the plasma membrane upon dexamethasone stimulation, and again, this effect was reversed by caspase inhibition (fig. S1B). No regulatory role of dexamethasone was detected in anti–Fas-induced extrinsic apoptosis of Jurkat T cells (fig. S1C). With respect to ferroptosis, research has been typically performed in HT1080 cells, but an effect of dexamethasone on ferroptosis progression has not been investigated to the best of our knowledge. We first examined whether dexamethasone alone induces cell death in HT1080 cells. Treatment of these cells for 20 to 40 hours with 20 μM dexamethasone (fig. S2A), and even ultrahigh concentrations of 100 μM for 50 hours (fig. S2B), did not cause any detectable cell death when compared to untreated controls. Next, we induced ferroptosis by inhibition of system Xc^−^ through 5 μM erastin. By flow cytometry, we detected significantly less annexin V/7AAD double-negative cells (living cells) and reciprocally significantly higher percentage of annexin V/7AAD double-positive cells in the erastin + dexamethasone–treated group as compared with the erastin only–treated group within the first 30 hours (Fig. 1, A and B). To directly assess the effect of dexamethasone on ferroptosis, we generated a three-dimensionally (3D) printed incubation chamber that contains two sides of one well separated by a glass slide (see fig. S3 and Materials and Methods for details). This chamber allowed us to perform time-lapse imaging with a single camera for the entire observation period of 30 hours following preincubation with vehicle or 1 μM dexamethasone. We added SYTOX green and annexin V to both sides of the chamber and stimulated the cells with 5 μM erastin. An earlier and more pronounced positivity for SYTOX green was detected in the dexamethasone-treated cells (Fig. 1C). Upon quantification, as many as 85% of cells exhibited a SYTOX-positive signal at 30 hours following 5 μM erastin + 1 μM dexamethasone treatment, while approximately only 40% of the cells were positive in the erastin-stimulated controls (Fig. 1C). Collectively, these data suggested that 1 μM dexamethasone significantly sensitizes HT1080 cells to erastin-induced ferroptosis.

We next set out to induce ferroptosis by the small-molecule RSL3, a well-characterized inhibitor of the glutathione peroxidase 4 (GPX4). In contrast to ferroptosis induction by erastin, dexamethasone pretreatment did not change the percentage of annexin V/7AAD–negative cells upon RSL3-induced ferroptosis induction (Fig. 1E). The difference between erastin- and RSL3-induced ferroptosis induction suggests that the dexamethasone-induced acceleration of ferroptosis is limited to the GSH regulatory part of the ferroptosis pathway. Theoretically, lower GSH levels might still influence GPX4 activity. To test this possibility in more detail, we investigated 36 hours of pretreatment with dexamethasone before RSL3 treatment (fig. S4A) and tested different concentrations of RSL3 (fig. S4B), especially sublethal concentrations up to 50 nM (fig. S4C). In conclusion, these data suggest that dexamethasone does not affect RSL3-induced ferroptosis.

### Glucocorticoids, but not aldosterone or dehydroepiandrostendione, sensitize to erastin-induced ferroptosis

**Fig. 2.**
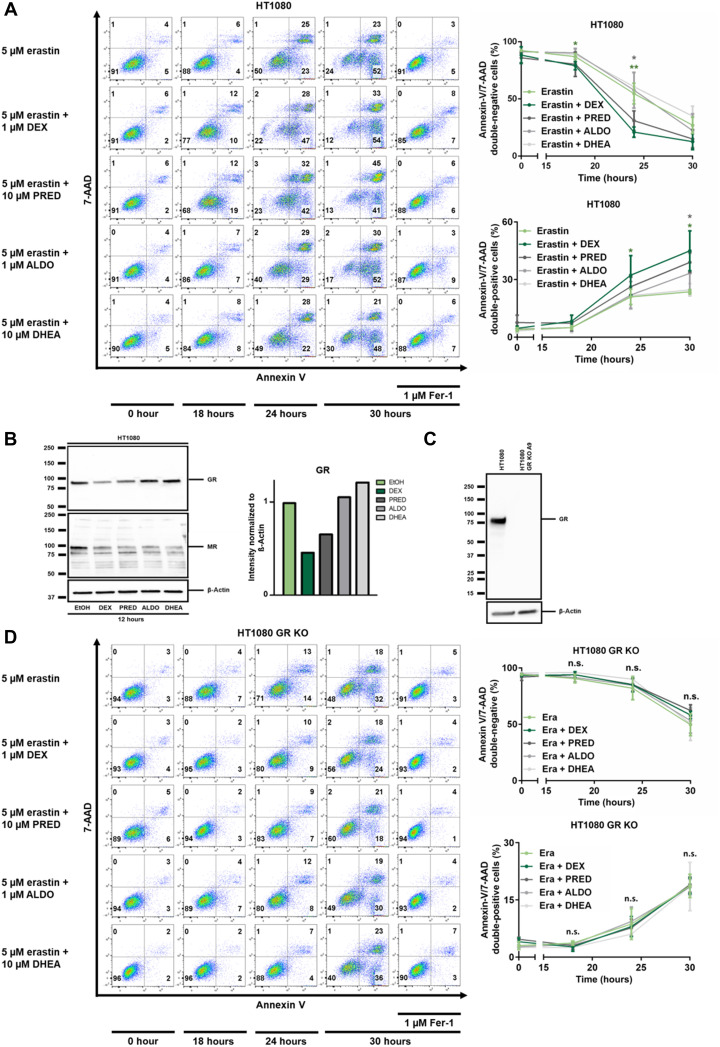
Steroid-induced sensitization to erastin-induced ferroptosis requires the GR 1. (**A**) HT1080 cells were treated with erastin, Fer-1, 1 μM dexamethasone (DEX), 10 μM prednisolone (PRED), 1 μM aldosterone (ALDO), and 10 μM dehydroepiandrostendione (DHEA) as indicated. Primary FACS plots and respective quantifications of indicated populations are demonstrated. Note the sensitization to ferroptosis induced by DEX and PRED, but not by ALDO and DHEA. (**B**) Assessment of GR and mineralocorticoid receptor (MR) down-regulation in response to indicated steroid hormones. β-Actin serves as a loading control. (**C**) CRISPR-Cas9–mediated knockout of GR from HT1080 cells, confirmed by Western blotting. (**D**) Experiment performed as described for (A), but using GR-knockout cells. Note the loss of sensitization to ferroptosis. All experiments shown are representative of at least three independent complete repetitions performed. The graphs show means ± SD. Statistical analysis was performed using Student’s *t* test for each time point. *P ≤ 0.05, **P ≤ 0.01, n.s., nonsignificant.

Given the potential redundancy between steroid hormones, we next asked whether other steroid hormones might phenocopy the dexamethasone effect on ferroptosis. While dexamethasone exhibited the strongest effect, prednisolone and, if at all only to a very minor extent, also aldosterone exhibited sensitization in this assay, while the coincubation with dehydroepiandrostendione (DHEA) had no effect (Fig. 2A). It is well known that treatment with glucocorticoids results in receptor down-regulation of the GR, and we confirmed this effect in ferroptosis-sensitive cells following stimulation with dexamethasone and prednisolone. In contrast, aldosterone and DHEA did not result in a similarly significant down-regulation of the GR. As an additional control, we investigated the expression levels of the mineralocorticoid receptor (MR), which is known to be down-regulated by aldosterone and found that also dexamethasone and prednisolone down-regulated the MR (Fig. 2B). In summary, these data suggested that the dexamethasone-induced sensitization to ferroptosis might be mediated through the GR.

### Dexamethasone-induced sensitization to ferroptosis requires the GR

Dexamethasone and other glucocorticoids were described to control cellular processes either transcriptionally through the GR or in a GR-independent manner. To test the role of the GR in our system, we stably transfected HT1080 cells with Cas9 and added guide RNAs to generate an HT1080 crKO (CRISPR knockout) of the *GR* (Fig. 2C). In contrast to control cells, deletion of the GR entirely reversed the sensitization to erastin-induced ferroptosis mediated by dexamethasone or prednisolone (Fig. 2D). These results demonstrate that glucocorticoid-induced sensitization to erastin-induced ferroptosis requires the GR.

### Dexamethasone treatment reduces GSH in HT1080 cells

**Fig. 3.**
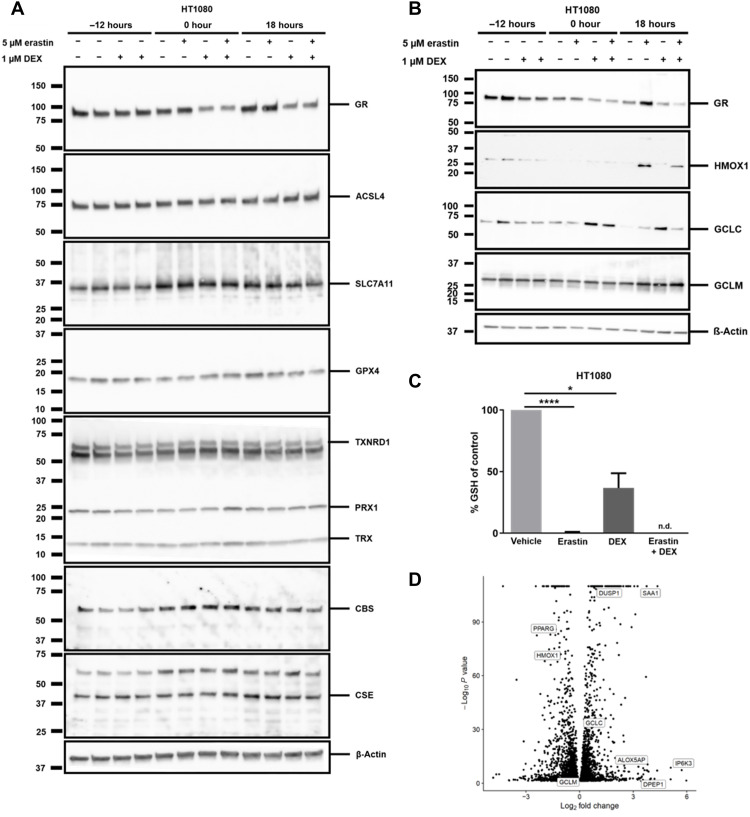
An unbiased bulk RNA-seq of parental HT1080 and GR-crKO cells reveals dexamethasone-induced genes involved in ferroptosis. (**A**) Protein expression of key players in ferroptosis 12 hours after treatment with or without 1 μM dexamethasone with subsequent treatment of induction of ferroptosis for 18 hours with erastin. β-Actin serves as a loading control. (**B**) Protein expression of HMOX1, GCLC, and GCLM 12 hours after treatment with or without 1 μM dexamethasone with subsequent treatment of induction of ferroptosis for 18 hours with erastin. β-Actin serves as a loading control. (**C**) GSH content in HT1080 cells treated with or without 1 μm dexamethasone before inducing ferroptosis with 5 μM erastin for 14 hours. (**D**) Volcano plot of differently regulated genes upon dexamethasone treatment in HT1080 cells. The bar graph shows means ± SD. Statistical analysis was performed using Student’s *t* test. *P ≤ 0.05, ****P ≤ 0.0001 (GR: glucocorticoid receptor, ACSL4: acyl-CoA synthetase long chain family member 4, SCL7A11: solute carrier family 7 member 11, GPX4: GSH peroxidase 4, TXNRD1: thioredoxin reductase 1, PRX1: peroxiredoxin 1, TRX: thioredoxin, CBS: cystathionine beta-synthase, CSE: cystathionine gamma lyase, HMOX1: heme oxygenase 1, GCLC: glutamate-cysteine ligase catalytic subunit, GCLM: glutamate-cysteine ligase modifier subunit, GSH: glutathione, n.d.: not detectable).

To understand how GR stimulation by dexamethasone influences erastin-induced but not RSL3-induced ferroptosis, we first looked at protein expression levels of known key players of the ferroptosis pathway. As demonstrated in Fig. 3A, no significant changes in protein expression of acyl-CoA synthetase long chain family member 4 (ACSL4), solute carrier family 7 member 11 (SLC7A11), GPX4, thioredoxin reductase 1 (TXNRD1), peroxiredoxin 1 (PRX1), thioredoxin (TRX), cystathionine beta-synthase (CBS), or cystathionine gamma lyase (CSE) were detected following dexamethasone stimulation. In addition, we investigated the protein expression levels of heme oxygenase 1 (HMOX1), glutamate-cysteine ligase catalytic subunit (GCLC), and glutamate-cysteine ligase modifier subunit (GCLM) (Fig. 3B). As expected, HOMX1 was up-regulated by erastin (*28*, *29*), while GCLC was up-regulated by dexamethasone. To gain further insight into the cellular redox state, we used liquid chromatography electrospray ionization quadrupole-time-of-flight (LC-ESI-QToF) mass spectrometry (MS) to accurately assess the level of GSH upon treatment with 1 μM dexamethasone (fig. S5A). As a GSH-depletion control, erastin was added as indicated in Fig. 3C. While erastin entirely depleted the GSH pool, dexamethasone resulted in a greater than 50% reduction of GSH content without significant numbers of cells undergoing actual ferroptosis (plasma membrane rupture; fig. S5B). This experiment explains why pretreatment with dexamethasone sensitizes to erastin-induced ferroptosis, while RSL3-induced ferroptosis remains unaltered, as the latter bypasses the need for GSH depletion upon ferroptosis induction. In line with this conclusion, β-mercaptoethanol also prevented cell death and GSH depletion in comparable settings (fig. S6, A and B).

### Dexamethasone induces DPEP1 expression

To identify potentially unknown regulators of ferroptosis sensitivity downstream of GR activation, we performed an unbiased bulk RNA-seq (fig. S7A). We confirmed accuracy of the investigated groups by principal component 1 (PC1)/PC2 variance (fig. S7B) and quality control assessments for RNA-seq accuracy (fig. S8, A and B) and assessed a hierarchical cluster of RNA-seq results (fig. S8C). In addition, we performed alignment statistics for RNA-seq analysis (fig. S8D). Moreover, we validated the GR knockdown in the RNA-seq analysis (fig. S8E). Among the most abundantly up- and down-regulated genes (data S1 and S2), several genes encoding for proteins previously associated with redox homeostasis appeared as demonstrated in a volcano plot in Fig. 3D. Among the top hits of up-regulated proteins, we decided to further validate DPEP1, also known as dehydropeptidase-I (DHP-I), microsomal dipeptidase (MDP), or renal dipeptidase (*30*). DPEP1 is a zinc-dependent metalloproteinase that has been shown to process antibiotics and hydrolyze a variety of peptides, including GSH breakdown products [cysteinylglycine (Cys-Gly)] (*31*). GSH was previously suggested to be involved in acute kidney injury (*32*).

### Knockdown of DPEP1 reverts dexamethasone-licensed sensitization to erastin-induced ferroptosis

**Fig. 4.**
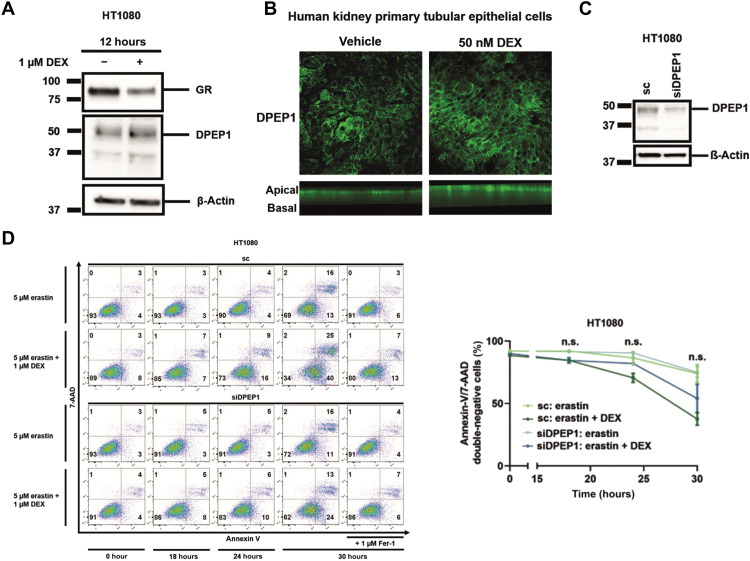
Dexamethasone-mediated sensitization to erastin-induced ferroptosis is mediated by DPEP1. (**A**) HT1080 cells were treated for 12 hours with dexamethasone before investigating protein expression levels of the GR and DPEP1. (**B**) Human kidney primary tubular epithelial cells (hTEC) were grown on Transwells and treated for 24 hours with dexamethasone before fluorescently labeling DPEP1. (**C**) HT1080 cells were treated 48 hours with siRNA against DPEP1, and knockdown efficacy was confirmed by Western blot. (**D**) HT1080 cells were treated with erastin and dexamethasone as indicated following the 48-hour pretreatment with an siRNA against DPEP1. Primary FACS plots and respective quantifications of indicated populations are demonstrated. The graphs show means ± SD. Statistical analysis was performed using Student’s *t* test for each time point. sc, scrambled; n.s., nonsignificant.

DPEP1 is up-regulated by treatment with 1 μM dexamethasone for 12 hours at the protein level (Fig. 4A). In keeping with this observation, DPEP1-labeled immunofluorescence in human primary kidney tubular epithelial cells cultured in Transwells exhibited an increase upon dexamethasone treatment while maintaining apical expression/polarity (Fig. 4B). In summary, these results confirm that dexamethasone indeed up-regulates protein expression of DPEP1. To test a functional role of DPEP1, we performed knockdown experiments in HT1080 cells and confirmed knockdown efficiency (Fig. 4C). As demonstrated in Fig. 4D, the knockdown of DPEP1 reverses dexamethasone sensitization to erastin-induced ferroptosis. However, as observed for the knockdown of DPEP1, the DPEP1 inhibitor cilastatin alone does not protect from erastin-induced ferroptosis (fig. S9, A to C).

### Dexamethasone accelerates the spontaneous lactate dehydrogenase release from freshly isolated murine renal tubules

**Fig. 5.**
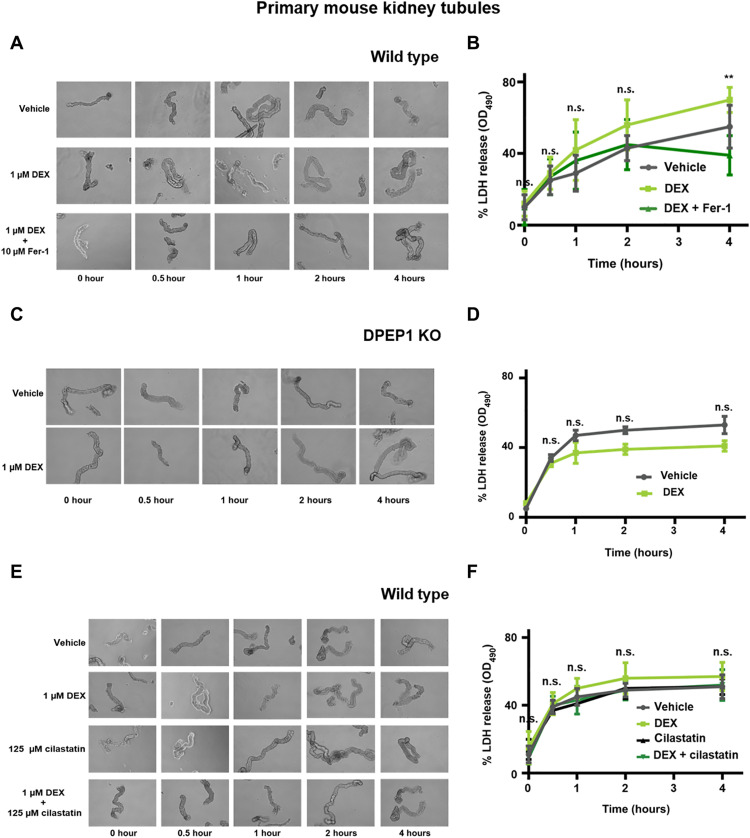
Ferroptosis in freshly isolated renal tubules is accelerated by dexamethasone in wild-type but not in DPEP1 knockout mice. (**A**) Representative images of freshly isolated murine kidney tubules undergoing spontaneous cell death in the presence of either vehicle or 1 μM dexamethasone in the presence or absence of 10 μM Fer-1. (**B**) LDH release of respective time points. (**C**) Representative images of freshly isolated murine kidney tubules from DPEP1 knockout mice undergoing spontaneous cell death in the presence of vehicle or 1 μM dexamethasone. (**D**) LDH release of respective time points. (**E**) Representative images of freshly isolated murine kidney tubules undergoing spontaneous cell death in the presence of either vehicle, 1 μM dexamethasone, 125 μm cilastatin, or dexamethasone and cilastatin. (**F**) LDH release of respective time points. The graphs show means ± SD. Statistical analysis was performed using Student’s *t* test for each time point. **P ≤ 0.01.

Ferroptosis drives spontaneous acute tubular necrosis in freshly isolated renal tubules (*33*). We used this ex vivo model (fig. S10) to assess the effect of dexamethasone on lactate dehydrogenase (LDH) release from renal tubules. Indeed, dexamethasone cotreatment led to significantly increased LDH release compared with vehicle-treated tubules, and this effect was reversed by the addition of ferrostatin-1 (Fer-1) (Fig. 5, A and B) or β-mercaptoethanol (fig. S11, A and B). Dexamethasone failed to accelerate LDH release from tubules isolated from DPEP1-deficient mice (Fig. 5, C and D). In keeping with a role of DPEP1 during the dexamethasone-induced sensitization, dexamethasone/cilastatin cotreated tubules did not exhibit higher LDH release levels as compared with vehicle-treated tubules (Fig. 5, E and F). Intriguingly, and in line with the data obtained from cell culture (fig. S9, B and C), cilastatin alone did not protect kidney tubules from LDH release. In summary, these data suggest a permissive pro-ferroptotic effect of dexamethasone mediated by the GR and DPEP1 (fig. S12).

## DISCUSSION

A growing body of evidence suggests the importance of ferroptosis in acute kidney injury and tubular necrosis (*22*, *24*, *33*–*39*). The molecular trigger of ferroptosis, however, still remains unclear, but intracellular lactate acidosis and a shift toward lower pH might facilitate intracellular Fenton reactions. Our data urge caution on the use of dexamethasone and, potentially, other steroids, as they may sensitize to tubular necrosis by ferroptosis. Trials using DPEP1 inhibitors to protect from acute kidney injury may appear far at the horizon given this first preclinical set of data. However, the clinical use of DPEP1 inhibitors upon treatment with carbapenems, such as imipenem-cilastatin, suggests sufficient safety at least in the class of DPEP1 inhibitors.

The DPEP1 inhibitor cilastatin is in clinical use as a combined therapeutic with β-lactam antibiotics to prevent DPEP1-mediated hydrolysis of drugs such as imipenem. Dipeptidases are well-established Cys-Gly cleaving enzymes that were demonstrated to affect intra- and extracellular concentrations of GSH by regulating GSH degradation (*40*). Dipeptidase mutant *Saccharomyces cerevisiae* cannot grow with GSH as the sole sulfur source (*41*). Likewise, human DPEP1, initially identified as a disulfide-linked homodimer that is glycosylphosphatidylinositol anchored to the renal brush border of the kidney (*42*), functions as a plasma membrane–bound zinc peptidase, which regulates renal GSH metabolism (*43*). DPEP1 is reported to have a subcellular localization on the brush border (luminal face) of the proximal tubular epithelium (*44*). As ferroptosis occurs in a non–cell autonomous manner (*24*, *45*), osmotic conditions outside of the plasma membrane, e.g., calcium gradients (*46*), may affect ferroptosis propagation.

DPEP1 and ferroptosis were identified in the pathogenesis of experimental contrast-induced acute kidney injury (CI-AKI) (*36*). We demonstrated a protective effect of the molecule necrostatin-1 (Nec-1) in a similar model (*47*). As Nec-1 is known to also inhibit ferroptosis (*22*), in retrospect, we consider it possible that the beneficial effects of Nec-1 on the CI-AKI model may be due to ferroptosis modulation rather than inhibition of receptor-interacting protein kinase 1 (RIPK1). Furthermore, a recent study identified single-nucleotide polymorphisms (SNPs) in close proximity to the DPEP1 gene to be frequently associated with AKI to chronic kidney disease progression in a large patient cohort (*48*). In addition, carnosine dipeptidase II (CNDP2) was demonstrated to protect cells under cysteine insufficiency by hydroliyzing GSH-related peptides (*49*). Further work is warranted to investigate the details of other dipeptidases in murine models of AKI and ferroptosis-related disorders.

Since the SARS-CoV-2 crisis, high-dose dexamethasone is applied to severely sick patients suffering from COVID-19. Given the millions of patients treated with this regimen, it is of paramount importance to better understand dexamethasone-associated side effects. The insight that dexamethasone lowers the threshold to ferroptosis through up-regulation of DPEP1 might be considered in future studies on the pathogenesis of steroid-induced untoward effects. In conclusion, we found the mechanism of a previously unrecognized ferroptosis-regulating function of dexamethasone.

## MATERIALS AND METHODS

### Reagents

**Table:** 

**REAGENT or RESOURCE**	**SOURCE**	**IDENTIFIER**
Antibodies		
GR (D6H2L) XP	Cell Signaling Technology	12041
Anti-FACL4 antibody (EPR8640)	Abcam	ab155282
PRX pathway (TRX, TXNRD1, and PRX1) WB cocktail	Abcam	ab184868
NR3C2 polyclonal antibody	Thermo Fisher Scientific	PA5-79761
CBS monoclonal antibody (mAB) (GT519)	Thermo Fisher Scientific	MA5-17273
Anti-gamma cystationase mouse mAB	Proteintech	60234-1-Ig
Anti-GPX4 antibody (EPNCIR144)	Abcam	ab125066
xCT/SLC7A11 (D2M7A) rabbit mAB	Cell Signaling Technology	12691
Recombinant anti–heme oxygenase 1 antibody (EPR1390Y)	Abcam	ab68477
Recombinant anti-GCLM antibody (EPR6667)	Abcam	ab126704
Recombinant anti-GCLC antibody (EP13475)	Abcam	ab190685
DPEP1 polyclonal antibody	Proteintech	12222-1-AP
β-Actin cell signaling	Cell Signaling Technology	3700S
Anti-mouse immunoglobulin (IgG); horseradish peroxidase (HRP)–linked antibody	Cell Signaling Technology	7076S
Anti-rabbit IgG; HRP-linked antibody	Cell Signaling Technology	7074S
Alexa Fluor 488 anti-rabbit IgG	Invitrogen	A21206
Compounds and chemicals		
Erastin [type 1 ferroptosis inducer (FIN)]	Sigma-Aldrich	E7781
RSL3 (type 2 FIN)	Selleck Chemicals	S8155
αFASL human, CH11	Merck Millipore	05-201
Fer-1	Merck Millipore	341494
2-Mercaptoethanol	Thermo Fisher Scientific	31350010
zVAD fmk	BD Biosciences	550377
Emricasan	Sigma-Aldrich	SML2227
Dexamethasone	Sigma-Aldrich	D4902
Prednisolone	Sigma-Aldrich	P6004
Aldosterone	Sigma-Aldrich	A9477
DHEA	Avanti/ Sigma-Aldrich	SKU 7000087P
Cilastatin	Sigma-Aldrich	C5743
Octyl-β-d-glucopyranosid	Sigma-Aldrich	O8001
Annexin V, Alexa Fluor 647 conjugate	Thermo Fisher Scientific	A23204
SYTOX Green	Life Technologies	S7020
7-AAD	BD Biosciences	559925
Annexin V–fluorescein isothiocyanate (FITC)	BD Biosciences	556420
Annexin V binding buffer	BD Biosciences	556454
ProLong Gold antifade	Invitrogen	P36391
LDH release assay	Promega	G1780
Bradford assay	Thermo Fisher Scientific	23225
ECL Prime Western Blotting System	Thermo Fisher Scientific	GERPN2232
Opti-MEM I Medium	Thermo Fisher Scientific	31985062
Lipofectamine RNAiMAX	Thermo Fisher Scientific	13778075
DPEP1 small interfering RNA (siRNA) (s4246)	Thermo Fisher Scientific	4392420
Silencer Select Negative Control #1 siRNA	Thermo Fisher Scientific	4390843

### Cell lines and cell culture

Human HT1080 cells were purchased from the American Type Culture Collection. HT1080 cells were cultured in Dulbecco’s modified Eagle’s medium (DMEM; Thermo Fisher Scientific) supplemented with 10% (v/v) fetal bovine serum (FBS; Thermo Fisher Scientific), penicillin (100 U/ml), and streptomycin (100 μg/ml) (Thermo Fisher Scientific). RS4;11 cells were purchased from the German Collection of Microorganisms and Cell Cultures (DSMZ) and cultured in α-MEM with ribo- and deoxyribonucleosides (Thermo Fisher Scientific) supplemented with 10% (v/v) FBS (Thermo Fisher Scientific), penicillin (100 U/ml), and streptomycin (100 μg/ml) (Thermo Fisher Scientific). Jurkat T cells were provided by A. Rösen-Wolff and cultured in RPMI 1640 medium (Thermo Fisher Scientific) supplemented with 10% (v/v) FBS (Thermo Fisher Scientific), penicillin (100 U/ml), and streptomycin (100 μg/ml) (Thermo Fisher Scientific). Murine thymocytes were isolated as described below and cultured in RPMI 1640 medium (Thermo Fisher Scientific) supplemented with 10% (v/v) FBS (Thermo Fisher Scientific), penicillin (100 U/ml), and streptomycin (100 μg/ml) (Thermo Fisher Scientific). All cells were cultured in a humidified 5% CO_2_ atmosphere.

### Cell culture of human proximal tubular epithelial cells

Nondiseased residual kidney tissue was obtained from medically indicated human nephrectomies. Kidney tissues were collected within 2 hours of surgery in Hanks’ balanced salt solution (HBSS) supplemented with penicillin-streptomycin (5 mg/ml) on ice. Human nephrectomy sample collection was approved by the Conjoint Health Research Ethics Board at the University of Calgary and Alberta Health Services. Following removal of the renal capsule, cortical tissue was carefully cut away and minced using aseptic techniques. After digestion in collagenase (1.5 mg/ml in HBSS) at 37°C for 60 min, samples were passed through serial filters from 200 to 45 μm to remove intact glomeruli and large cellular debris. Samples were then plated on plastic cell culture plates at 37°C for 90 min in K1 culture medium DMEM/F12 containing 10% FBS, 1% penicillin-streptomycin, prostaglandin E1 (125 ng/ml), l-thyroxine (1.8 μg/ml), hydrocortisone (3.38 ng/ml), insulin-transferrin-sodium selenite supplement (2.5 mg/ml), and epithelial growth factor (10 ng/ml; Sigma-Aldrich). Human proximal tubular epithelial cells (HPTC) were collected and plated onto collagen IV–coated cell culture plates. To maintain the epithelial phenotype, HPTC were used less than two passages.

### Generation of GR-deficient HT1080 cells

We used a two-vector CRISPR-Cas9 approach. In the first step, HT1080 cells were transduced with viral supernatant containing the lentiCas9-Blast vector and packing plasmids (pMDLg/pRRE, pRSV-Rev, and pMD2.G). Cells were selected using Blasiticidin (5 μg/ml; InvivoGen). After outgrowth of single cells, Cas9 expression was verified by Western blotting. Guide RNAs targeting the GR (GAACACTGGTCGACCTATTG) were cloned into the lentiGuide-Puro vector. Subsequently, Cas9-expressing HT1080 cells were transduced with viral supernatant containing the lentiGuide-Puro vector and packing plasmids. After selection with puromycin (1 μg/ml; InvivoGen), knockout efficacy was determined on protein level in single cells.

### siRNA-mediated knockdown of DPEP1

HT1080 cells were plated in a petri dish in 15 ml of antibiotic-free medium. After 24 hours, 180 pmol RNAi and 24 μl of Lipofectamine (Thermo Fisher Scientific) were each mixed in 1.5 ml of Opti-MEM I Medium (Thermo Fisher Scientific) without serum, combined, and incubated at room temperature for 20 min before dropping the mixture on the cells. The following day, cells were harvested, plated into six-well plates, and cell death assays were performed as described below. Knockdown efficacy was determined on protein level.

### Western blotting

Cells were lysed in ice-cold 50 mM tris-HCl (pH 7.5), 150 mM NaCl, 1% NP-40, 5 mM EDTA supplemented with PhosSTOP (Merck), cOmplete (Merck), and 1 mM phenylmethylsulfonyl fluoride (PMSF) for 30 min on ice. To determine DPEP1 protein levels, cells were lysed in ice-cold 50 mM tris-HCl (pH 8), 150 mM NaCl, 0.5% sodium desoxychelat, 0.1% SDS supplemented with PhosSTOP (Merck), cOmplete (Merck), 1 mM PMSF, and 100 mM Octyl-β-d-glucopyranosid for 30 min on ice. Insoluble material was removed by centrifugation (14,000*g*, 30 min, 4°C). Protein concentration was determined using a commercial bicinchoninic acid assay kit according to the manufacturer’s instructions (Thermo Fisher Scientific). Equal amounts of protein (typically 25 μg per lane) were resolved on a 4 to 15% gradient SDS–polyacrylamide gel electrophoresis gel and transferred to a polyvinylidene difluoride membrane (Bio-Rad). After blocking for 1 hour at room temperature, primary antibody incubation was performed at 4°C overnight. Secondary antibodies (anti-mouse, HRP-linked antibody, and anti-rabbit, HRP-linked antibody, Cell Signaling Technology) were applied at concentrations of 1:5000. Proteins were then visualized by enhanced chemiluminescence (Amersham Biosciences).

### Cell death assays

Ferroptosis was induced using established FINs—type 1 FIN: erastin (Sigma-Aldrich), or type 2 FIN: RSL3 (Selleckchem). Cells were seeded in six-well plates or petri dishes and pretreated for 12 hours with 1 μM dexamethasone before induction of ferroptosis. Unless otherwise indicated, we used 5 μM erastin and 1.13 μM RSL3. After indicated time points, cells were collected and prepared for flow cytometry, immunoblotting, or live-cell imaging.

### Flow cytometry

Cells were harvested, and the pellets were washed twice in phosphate-buffered saline (PBS) and stained with 5 μl of 7-AAD (BD Biosciences) and 5 μl of annexin V–FITC (BD Biosciences) added to 100 μl of annexin V–binding buffer (BD Biosciences). After 15 min, cells were recorded on the LSR II with the FACS Diva 6.1.1 software (BD Biosciences) and subsequently analyzed with the FlowJo v10 software (Tree Star). The flow cytometry procedure was supported by the Flow Cytometry Core Facility of the Center for Molecular and Cellular Bioengineering (CMCB) Technology Platform at Technische Universität (TU) Dresden.

### LDH release assay

The LDH release of cells or of freshly isolated kidney tubules was measured according to the manufacturers’ instructions at indicated time points. In brief, an aliquot of the supernatant was taken, and lysis solution was added for 45 min to induce maximal LDH release before another aliquot of the supernatant was taken. Subsequently, the supernatants were incubated with CytoTox 96 reagent for 15 min, protected from light at room temperature before adding stop solution. Absorbance was measured at 490 nm.

### Mass Spectrometry (LC-ESI-QToF)

Cells were placed on ice and washed with 0.9% NaCl before covering them with −80°C cold quenching buffer (80% LC-MS grade methanol) followed by a 20-min incubation time at −80°C. Subsequently, cells were scraped and centrifuged at 14,000*g*, 4°C for 10 min. The supernatant was centrifuged at 14,000*g*, 4°C for 10 min again before freezing it at −80°C until further analysis. For the MS-based analysis using an Aquity I-class ultra performance LC system (Waters) coupled to a high-resolution QToF-MS (Vion IMS QToF, Waters), 200-μl aliquots of the aforementioned cell lysates were dried down in a vacuum-assisted centrifuge for at least 2.5 hours and afterward reconstituted in 100 μl of initial mobile phase (10/90% water/acetonitrile containing 0.1% formic acid). For chromatographic separation, a Waters ACQUITY UPLC BEH column (2.1 × 100 mm, 1.7 μm) at 45°C and a gradient of mobile phases A (water/0.1% formic acid) and B (acetonitrile/0.1% formic acid) was used. Calibrators and samples (7.5 μl), kept at 4°C in the autosampler, were injected into the LC-QToF-MS system at a flow rate of 0.4 ml/min. Directly after injection at 10% mobile phase A, proportions of A linearily increased to 20% at 2.0 min to 48% at 4.0 min and further to 90% at 5.0 min. After a hold for 1 min at 90% mobile phase A, the gradient returned to initial conditions at 6.3 min, followed by another 1.2 min for column reequilibration. For identification and quantification of GSH, a single standard of GSH was purchased from Sigma-Aldrich (Munich, Germany). After disolvation and respective dilution in mobile phase, the GSH standard was injected into the LC-QToF-MS to determine analyte-specific characteristics, such as LC retention time (3.30 min), accurate mass [(M + H^+^) = 308.0915 Da], and the collision cross section (CCS) value (168.06 Å^2^), a metabolite-specific measure of ion mobility. GSH in calibrators and samples was detected by using the high-definition MS^E^ data acquisition mode that includes ion mobility separation and determination of CCS data, and accurate precursor and respective fragment ion masses of all ions. Positive electrospray ionization source parameters were set to 1.0 kV for capillary voltage, to 120° and 550°C for source and desolvation temperatures, respectively, as well as to respective cone- and desolvation gas flows at 50 and 900 liter/hour. Data were evaluated by using Waters with UNIFI Software package (version 1.9.4.053).

### Immunofluorescence microscopy

HPTC (see above) were cultured on collagen-coated coverslips and treated with dexamethasone as indicated. Cells were fixed with 4% paraformaldehyde and incubated with NH_4_Cl (50 μM) to quench free aldehyde groups. Cells were blocked in 3% bovine serum albumin (BSA) before incubation with a primary DPEP1 antibody overnight at 4°C. Cells were washed in PBS before incubation in secondary fluorescent antibody (Alexa Fluor 488 anti-rabbit IgG, A21206, Invitrogen). Coverslips were mounted onto slide in ProLong Gold antifade (P36391, Invitrogen) containing 4′, 6-diamidino-2-phenylindole. Images were acquired with a confocal fluorescence microscope (Olympus IX-70) using the Fluoview1000 software (Olympus, Tokyo, Japan).

### 3D printing of a double chamber for simultaneous live-cell imaging

For improved comparability of dexamethasone-treated and untreated cells, we aimed to design a two-well system. This allows for direct comparison of differentially treated cells using the same fluorescence camera. The used chambers consist of a border including a retainer for a glass wall and a glass wall, which is separating the two chambers. The border was printed out of a silicone elastomer (SE 1700, Dow Corning) with the 3DDiscovery bioprinter from RegenHU using a conical nozzle with an inner diameter of 250 μm. Print layouts were developed in the BIOCAD software (RegenHU). The printing speed and extrusion pressure were adjusted to get the thickness of the printed line around 500 μm (these parameters depend on the age of silicone). The height of the border was 8 mm.

The chambers’ border was printed on top of a silanized microscope slide and cured at 100°C for 30 min. A line of SE1700 was printed in the center of the chambers using higher printing speed, thus making it much thinner than the border of the chambers (around 200 μm). SE1700 was applied to the retainer manually (via a syringe with a conical needle) to bond the glass wall. For the glass wall, a coverslip (20 mm by 20 mm; thickness, 0.12 mm) was cut to a size of 8 mm by 20mm, cleaned with ethanol, and treated with air plasma for 10 s using a cold-plasma generator Piezobrush PZ2-i equipped with a nearfield nozzle from Relyon Plasma. Then, the glass wall was inserted into the retainer and pushed down until it was in direct contact with the thin line of SE1700. This structure was again cured at 100°C for 30 min. The structure was removed from the microscope slide, and its bottom side as well as the inside of a six-well plate was treated with air plasma for around 10 s (using Piezobrush PZ2-i). Then, the structure was attached to a well of the six-well plate and kept at 60°C for 2 hours for bonding. In some cases, certain places of the border were poorly bonded to the surface. These places were sealed with SE1700 manually, and then the structure was placed again to 60°C for 4 hours.

### Time-lapse imaging

Time-lapse imaging was performed using a 2.5×/0.12 Fluar objective for the custom-made 3D-printed well plated with HT1080 cells on an Axiovert 200M equipped with a large incubation chamber (37°C), 5% CO_2_, and humidity control. Transmitted light and fluorescent images (LED 475-nm Spectra X light source, emission filter BP 525 to 550 and LED 631-nm Spectra X light source, emission filter BP 663 to 738) were acquired using an Orca flash 4.0 camera. The protocol used for staining was adapted from Wallberg *et al.* (*50*). HT1080 cells were plated in the above-described double chamber in DMEM without phenol red (Thermo Fisher Scientific) and pretreated for 12 hours with 1 μM dexamethasone or vehicle. Subsequently, 5 μM erastin was added, as well as 0.5 μM SYTOX Green (Life Technologies), annexin V (5 μg/ml), Alexa Fluor 647 conjugate (Thermo Fisher Scientific), and 2.5 mM CaCl_2_. The live-imaging procedure was supported by the Light Microscopy Facility, a core facility of the CMCB Technology Platform at TU Dresden.

### RNA-seq analysis

RNA was isolated using the RNeasy Mini Kit (QIAGEN). For quantification of gene abundances of the processed paired-end RNA-seq reads, a STAR (*51*) (version 2.7.6a) index was created on the basis of the human hg38 transcriptome. Gene abundances were quantified with RSEM (*52*) (version 1.3.1) using default settings. The estimated gene abundances were imported to R with tximport (*53*), and genes that had summed counts below 10 across all samples were removed from the downstream analysis. For outlier detection, gene counts were normalized with varianceStabilizingTransformation (blind = TRUE) from the DESeq2 (*54*) package (version 1.28.1), and a principal components analysis (PCA) was performed with RunPCA (ntop = 2000). Hierarchical clustering based on Euclidean distances was performed on the variance stabilized data and visualized with heatmap.2. Differential gene expression analysis was performed with DESeq using a log_2_ fold change threshold of 1 and false discovery rate <0.05. Significantly differentially expressed genes were visualized in volcano plots.

### Mice

Male mice (8 to 12 weeks old) were cohoused with two to five mice per cage in IVCs (individually ventilated cages) in our facility at the Medizinisch-Theoretisches Zentrum at the Medical Faculty of the Technical University of Dresden (TU Dresden). All wild-type mice (C57Bl/6N) were initially provided by Charles River, Sulzfeld, Germany, at the age of 6 to 7 weeks. DPEP1 knockout mice were described previously (*55*). All experiments were performed according to German animal protection laws and were approved by ethics committees and local authorities of Dresden (Germany).

### Isolation of murine thymocytes

After removal of thymi, organs were placed on a 100 μm cell strainer. Using a plunger of a 3-ml syringe, thymi were pressed through the cell strainer and rinsed with RPMI 1640 medium (Thermo Fisher Scientific). Thymocytes were washed twice before dead cells were removed by Ficoll-Paque PLUS (VWR). Subsequently, cells were counted, plated in 24-well plates, and treated with vehicle, 1 μM dexamethasone with or without 5 μM emricasan for the indicated time points.

### Isolation of primary murine renal tubules

Primary murine renal tubules were isolated following a modified previously published protocol (*33*). In detail (demonstrated in fig. S7), murine kidneys were removed, washed with PBS, and decapsulized and sliced in four to five slices. Kidney slices of each kidney were transferred in 2-ml reaction tube containing collagenase type II (2 mg/ml) in incubation solution [trypsin inhibitor (48 μg/ml), DNAse I (25 μg/ml), 140 mM NaCl, 0.4 mM KH_2_PO_4_, 1.6 mM K_2_HPO_4_ · 3 H_2_O, 1 mM MgSO_4_ · 7 H_2_O, 10 mM CH_3_COONa · 3 H_2_O, 1 mM α-ketoglytarate, and 1.3 mM Ca-gluconate] and digested for 5 min at 37°C, 850 rpm. Because of the presence of damaged tubules, the first resulting supernatant was discarded, and 1 ml of collagenase type II (2 mg/ml) in incubation solution was added to the kidney slices and digested for 5 min at 37°C, 850 rpm. The supernatant was collected and transferred in a 2-ml reaction tube containing 1 ml of ice-cold sorting solution (0.5 mg/ml BSA in incubation solution). The reaction tubes were left on ice for the tubules to precipitate. The supernatant was removed, and the tubules were washed twice with ice-cold incubation solution. Once the tubules precipitated, the supernatant was removed, and ice-cold sorting solution was added (the volume was adjusted depending on the number of samples needed for the experiment). Tubules were distributed in a 24-well plate containing DMEM F-12 nutrient mixture without phenol red (DMEM/F12, Thermo Fisher Scientific), supplemented with recombinant human insulin (0.01 mg/ml), human transferrin (5.5 μg/ml), sodium selenite (Na_2_SeO_3_, 0.005 μg/ml) (ITS without linoleic acid, Sigma-Aldrich), 50 nM hydrocortisone, penicillin (100 U/ml), and streptomycin (100 μg/ml) (Thermo Fisher Scientific).

### Statistical analysis

Statistical analyses were performed with Prism 8 (GraphPad software, San Diego, CA, USA) using Student’s *t* test. Data were considered significant when *P ≤ 0.05, **P ≤ 0.01, ***P ≤ 0.001, or ****P ≤ 0.0001.

## Notes

View/request a protocol for this paper from *Bio-protocol*.
