# Supplementary material for: Dexamethasone sensitizes to ferroptosis by glucocorticoid receptor–induced dipeptidase-1 expression and glutathione depletion
Source: Sci Adv. 2022 Feb 2;8(5):eabl8920. doi: 10.1126/sciadv.abl8920 (PMC8809683; doi:10.1126/sciadv.abl8920)
Supplement: Supplementary file 1 — Figs. S1 to S12 Legends for data S1 and S2 Legends for movies S1 and S2 [file sciadv.abl8920_sm.pdf]

Science Advances
AAAS

Supplementary Materials for

Dexamethasone sensitizes to ferroptosis by glucocorticoid receptor–induced dipeptidase-1 expression and glutathione depletion

Anne von Mässenhausen, Nadia Zamora Gonzalez, Francesca Maremonti, Alexia Belavgeni, Wulf Tonnus, Claudia Meyer, Kristina Beer, Monica T. Hannani, Arthur Lau, Mirko Peitzsch, Paul Hoppenz, Sophie Locke, Triantafyllos Chavakis, Rafael Kramann, Daniel A. Muruve, Christian Hugo, Stefan R. Bornstein, Andreas Linkermann*

*Corresponding author. Email: andreas.linkermann@ukdd.de

Published 2 February 2022, *Sci. Adv.* **8**, eabl8920 (2022)
DOI: 10.1126/sciadv.abl8920

The PDF file includes:

Figs. S1 to S12
Legends for data S1 and S2
Legends for movies S1 and S2

Other Supplementary Material for this manuscript includes the following:

Data S1 and S2
Movies S1 and S2

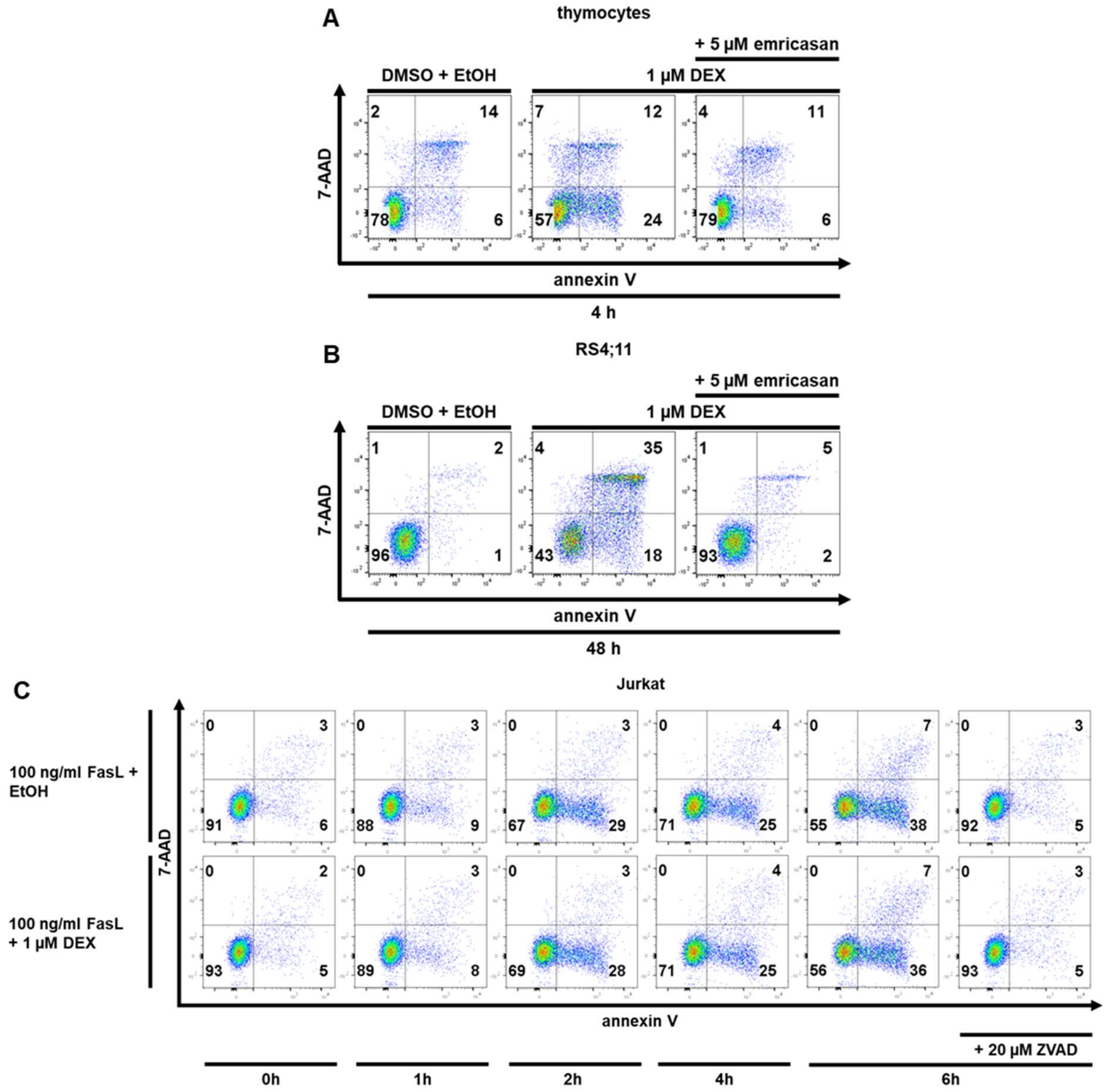

Fig. S1: Dexamethasone pretreatment does not affect Fas-induced apoptosis. (**A**) Thymocytes were stimulated with 10 µM dexamethasone for 4 hours. Annexin V / 7AAD double positivity was assessed by FACS analysis. (**B**) RS4;11 cells were treated with dexamethasone for 48 hours. Note the complete reversal of the annexin V / 7AAD-double positivity by addition of the caspase-inhibitor emricasan. (**C**) Co-incubation with dexamethasone does not affect Fas Ligand-induced apoptosis of Jurkat T cells.

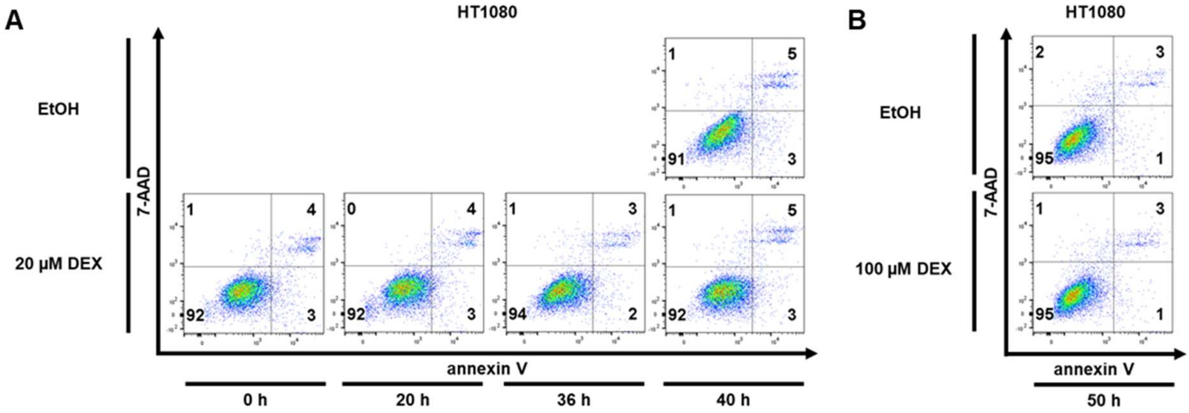

Fig. S2: High concentrations of dexamethasone do not result in any detectable cell death of HT1080 cells. (**A**) HT1080 cells were incubated with different concentrations of dexamethasone over time, as indicated. (**B**) Stimulation of HT1080 cells for 50 hours with 100µM dexamethasone does not affect positivity for annexin V or 7AAD.

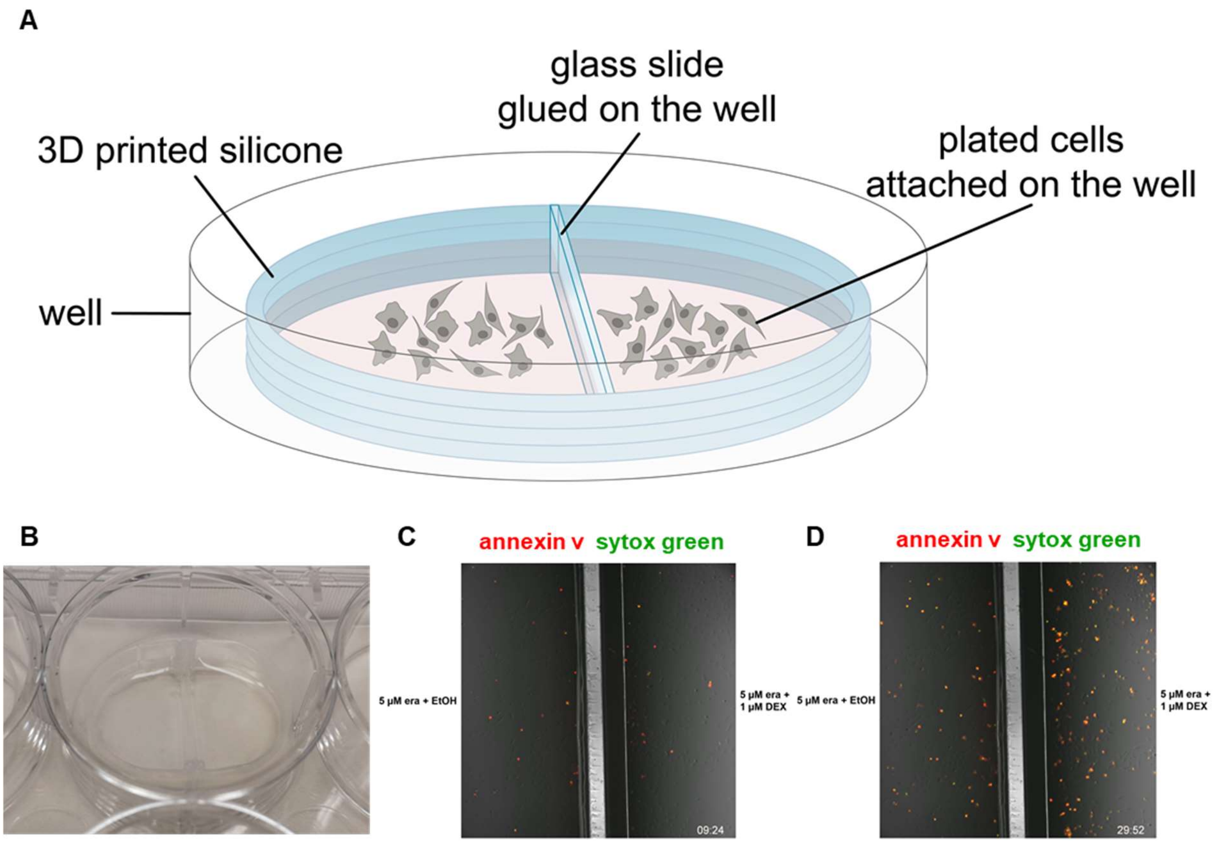

Fig. S3: Three dimentional (3D)-printing of a double chamber allows detection of accelerated ferroptosis in HT1080 cells upon co-stimulation with dexamethasone. (A) Cartoon of the 3 D printed chamber for time lapse analysis (corresponds to Fig. 1D and 1E). (**B**) Fotograph of the 3D-printed double chamber. (**C, D**) Still images taken at indicated times of the time lapse videos obtained from dexamethasone-treated HT1080 cells stimulated with erastin.

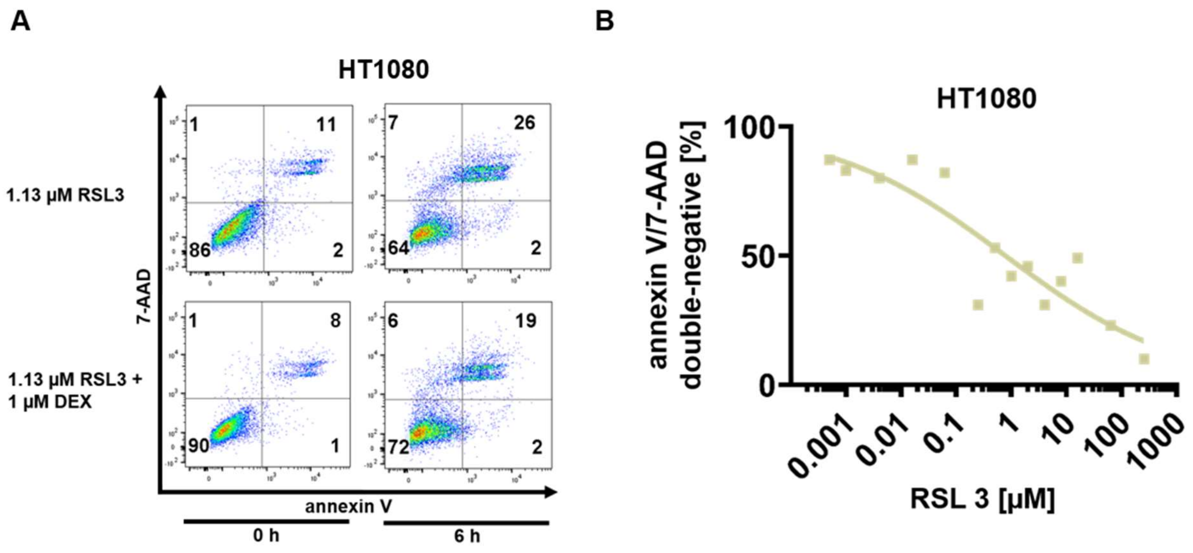

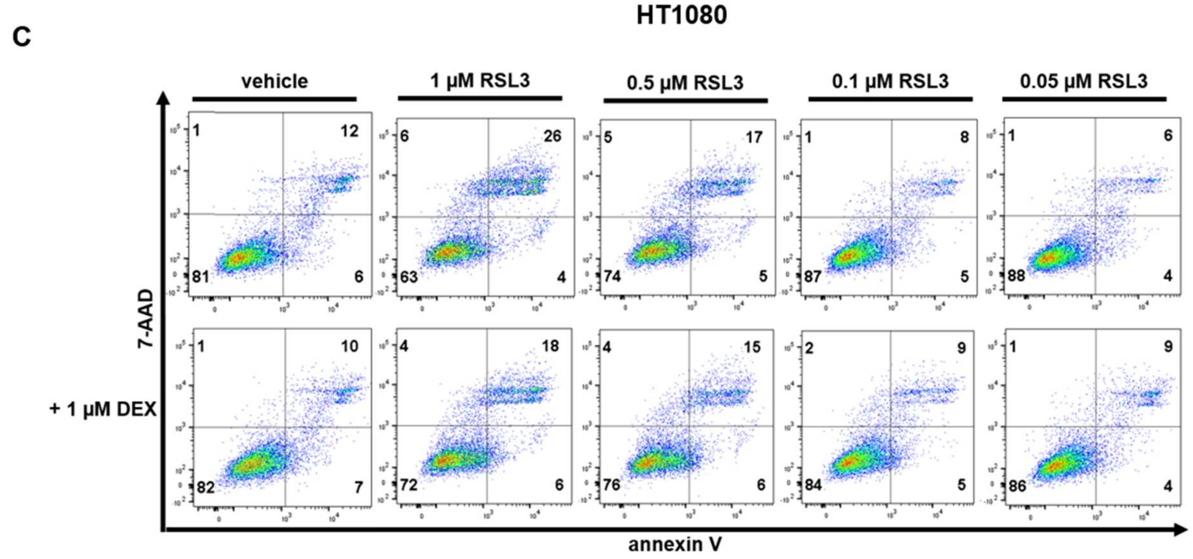

Fig. S4: Dexamethasone does not sensitize to RSL3-induced ferroptosis (A) HT1080 cells were treated for indicated times with 1.13 µM RSL3 with or without pre-treatment of 1 µM dexamethasone for 36 hours. 7-AAD and annexin V were read out by FACS. (B) HT1080 cells were treated with indicated amounts of RSL3 for 6 hours. 7-AAD and annexin V were read out by FACS and % of annexin V/7-AAD double negative cells are shown. (C) HT1080 cells were treated for 6 hours with indicated amounts of RSL3 with or without pre-treatment of 1 µM dexamethasone for 12 hours. 7-AAD and annexin V were read out by FACS.

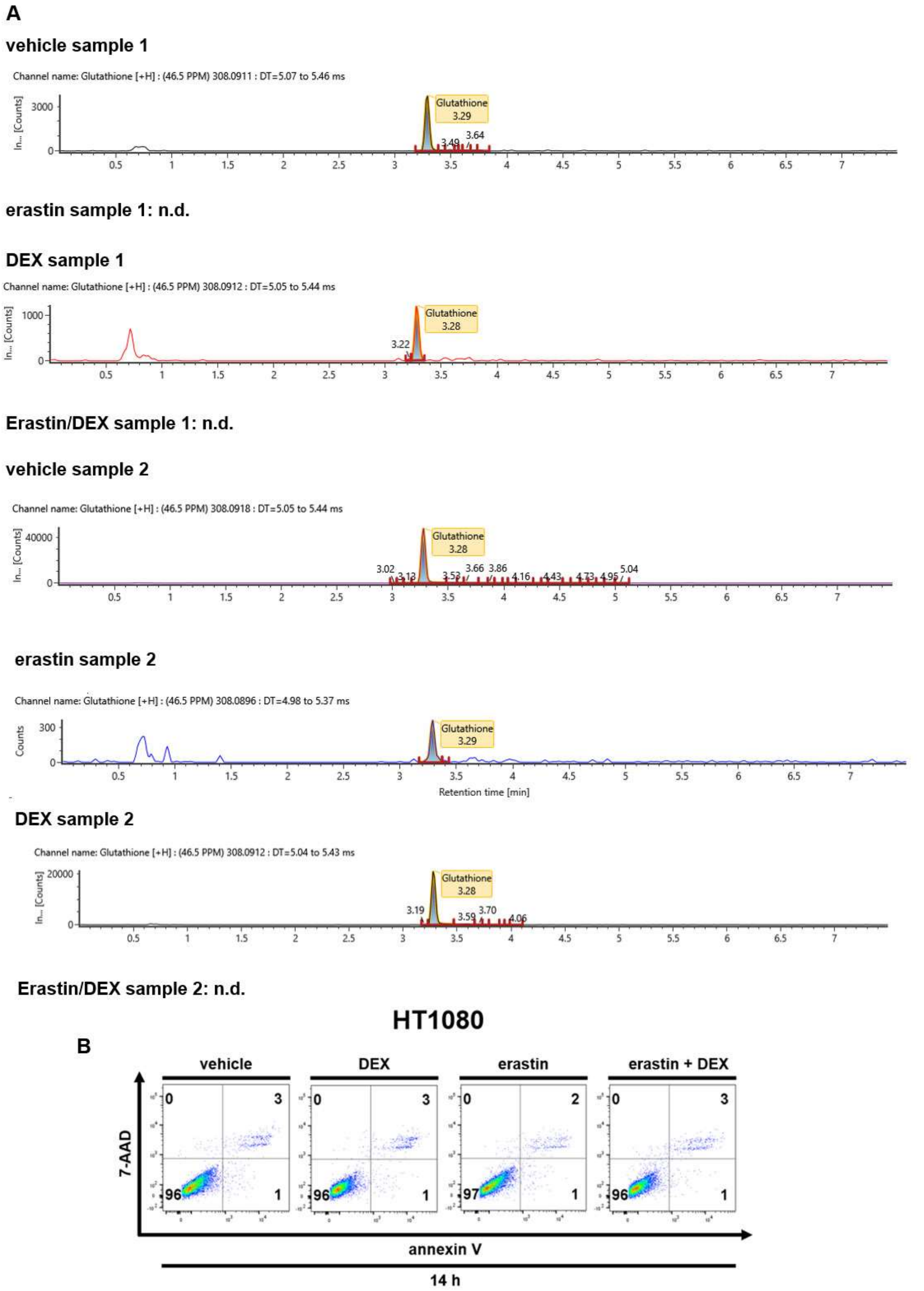

Fig. S5: Mass spectrometry (LC-ESI-QToF). (**A**) Analytes were detected in accurate mass screening and ion mobility separation mode using positive electrospray ionization. Acquired LC-ESI-QToF data was evaluated with UNIFI Software Version 1.9.4.053. Note the differentially labelled Y axes of each plot as indicated. (**B**) Control HT1080 cells of the experiments demonstrated in Fig. 3B and (**A**) were pre-treated with 1 µM dexamethasone for 12 hours before induction of ferroptosis with 5 µM erastin for 14 hours. Samples were collected as detailed in the material and methods section and subjected to LC-MS. (n.d. not detectable).

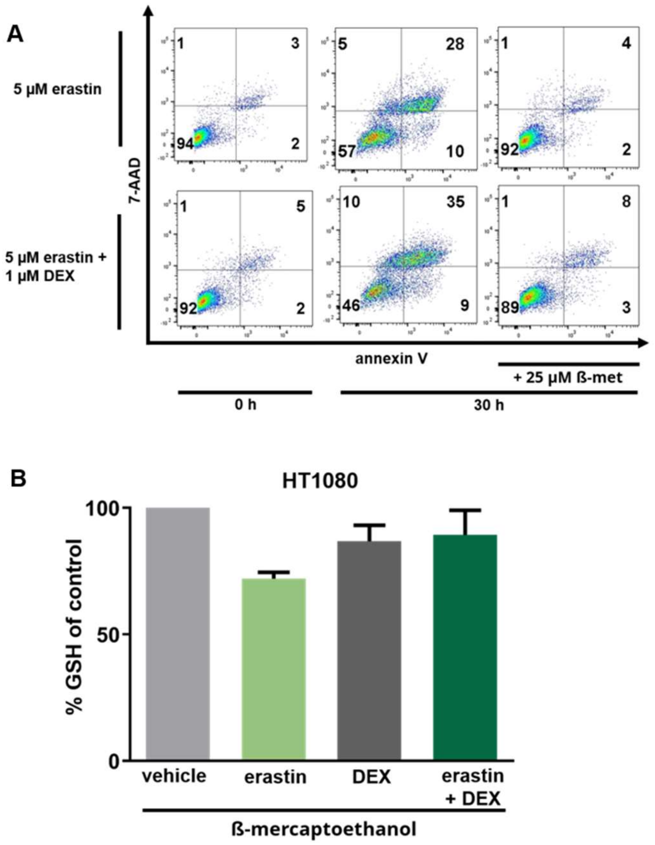

Fig. S6: ß-mercaptoethanol protects from the combined treatment of erastin and dexamethasone
(**A**) HT1080 cells were treated for indicated times with 5 µM erastin and 25 µM ß-mercaptoethanol (ß-met) with or without pre-treatment of 1 µM dexamethasone for 12 hours. 7-AAD and annexin V were read out by FACS. (**B**) Glutathione (GSH) content in HT1080 cells treated with or without 1 µm dexamethasone before inducing ferroptosis with 5 µM erastin for 14 hours with cotreatment of 25 µM ß-mercaptoethanol. The bar graph shows mean +/- SD. GSH: glutatione

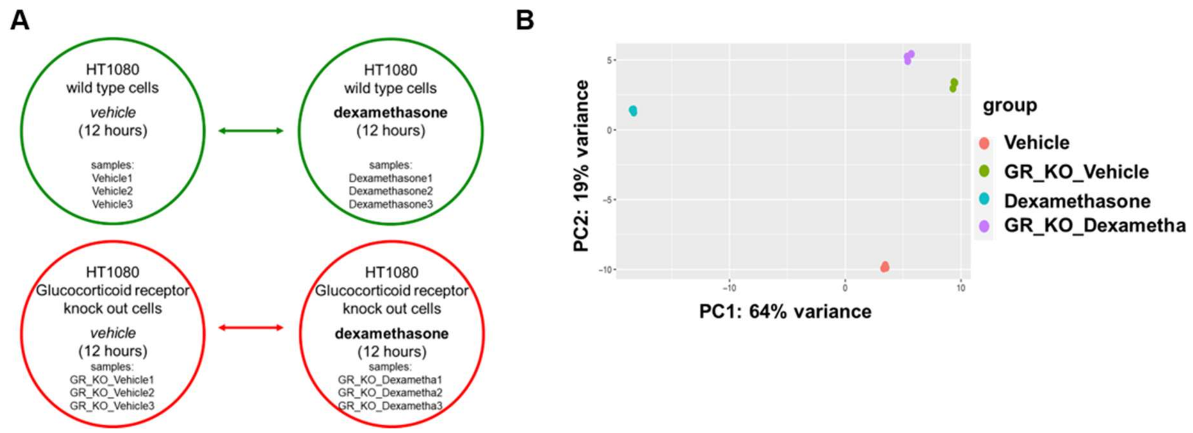

Fig. S7: Experimental setup for unbiased bulk RNA sequencing analysis. (**A**) HT1080 control cells or GR knockout cells were treated with 1 µM dexamethasone for 12 hours before isolating RNA. (**B**) Confirmation of accuracy of the investigated groups by PC1/PC2 variance.

A

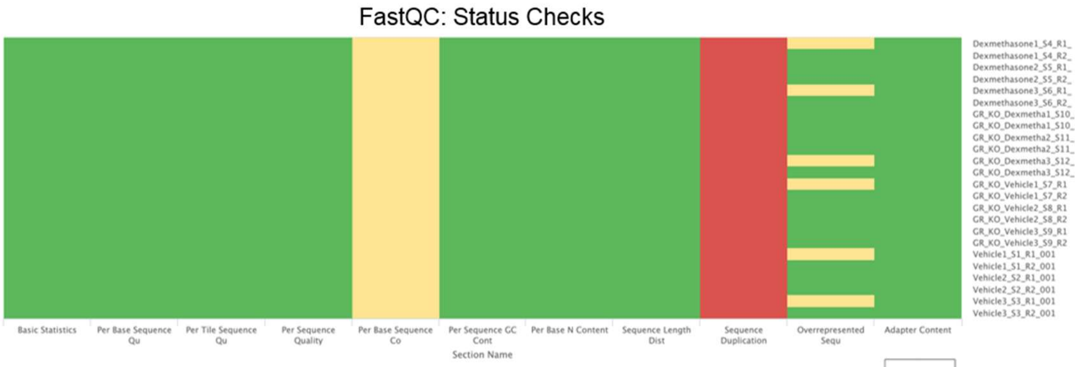

B

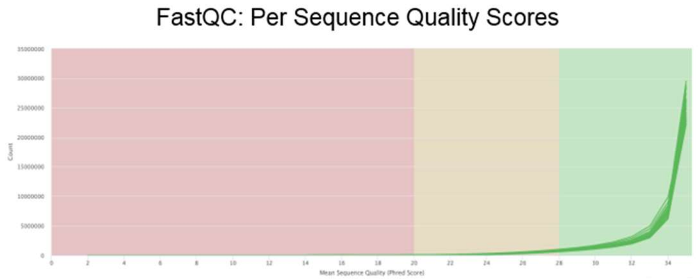

C

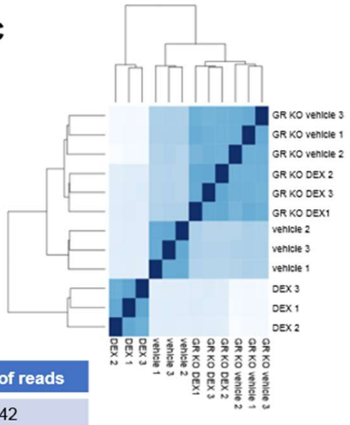

D

Sample	Map (%)	Unique Map (%)	Total number of reads
**Vehicle 1**	97,5	90,6	38,788,242
**Vehicle 2**	97,5	90,8	47,112,953
**Vehicle 3**	97,5	90,9	43,903,297
**Dexamethasone 1**	97,7	91,1	49,261,154
**Dexamethasone 2**	97,3	90,7	41,021,818
**Dexamethasone 3**	97,6	90,9	48,329,355
**GR_KO_Vehicle1**	97,2	90,6	44,986,611
**GR_KO_Vehicle2**	97,0	90,3	40,864,021
**GR_KO_Vehicle3**	97,5	90,8	50,852,228
**GR_KO_Dexmetha1**	97,0	90,5	45,654,577
**GR_KO_Dexmetha2**	97,2	90,6	43,415,781
**GR_KO_Dexmetha3**	97,2	90,6	44,880,088

E

Sample	Exon 1	Exon 2	Exon 3	Exon 4	Exon 5	Exon 6	Exon 7	Exon 8	Exon 9
**Vehicle 1**	13	**74**	14	9	20	14	12	12	42
**Vehicle 2**	12	**75**	13	10	22	13	13	14	47
**Vehicle 3**	12	**75**	14	11	21	13	12	11	46
**Dexamethasone 1**	10	**54**	9	7	14	9	8	8	30
**Dexamethasone 2**	10	**54**	10	7	15	10	9	8	31
**Dexamethasone 3**	10	**51**	9	7	14	9	7	7	30
**GR_KO_Vehicle1**	20	**43**	17	14	28	18	15	15	58
**GR_KO_Vehicle2**	17	**36**	15	11	24	17	15	16	57
**GR_KO_Vehicle3**	20	**41**	17	14	30	19	17	16	54
**GR_KO_Dexmetha1**	17	**37**	17	13	26	17	15	16	58
**GR_KO_Dexmetha2**	17	**36**	15	13	26	16	15	15	53
**GR_KO_Dexmetha3**	15	**31**	13	12	23	16	14	14	53

Fig. S8: An unbiased bulk RNA sequencing of parental and GR-crKO cells reveals dexamethasone-induced genes involved in ferroptosis. (**A**) Quality control assessments for RNAseq accuracy. (**B**) Alignment statistics for RNAseq analysis. (**C**) Validation of the glucocorticoid receptor knockdown in the RNAseq analysis. (**D**) Hierachial cluster of RNAseq results. (**E**) Differnetial gene expression profile of the RNAseq analysis.

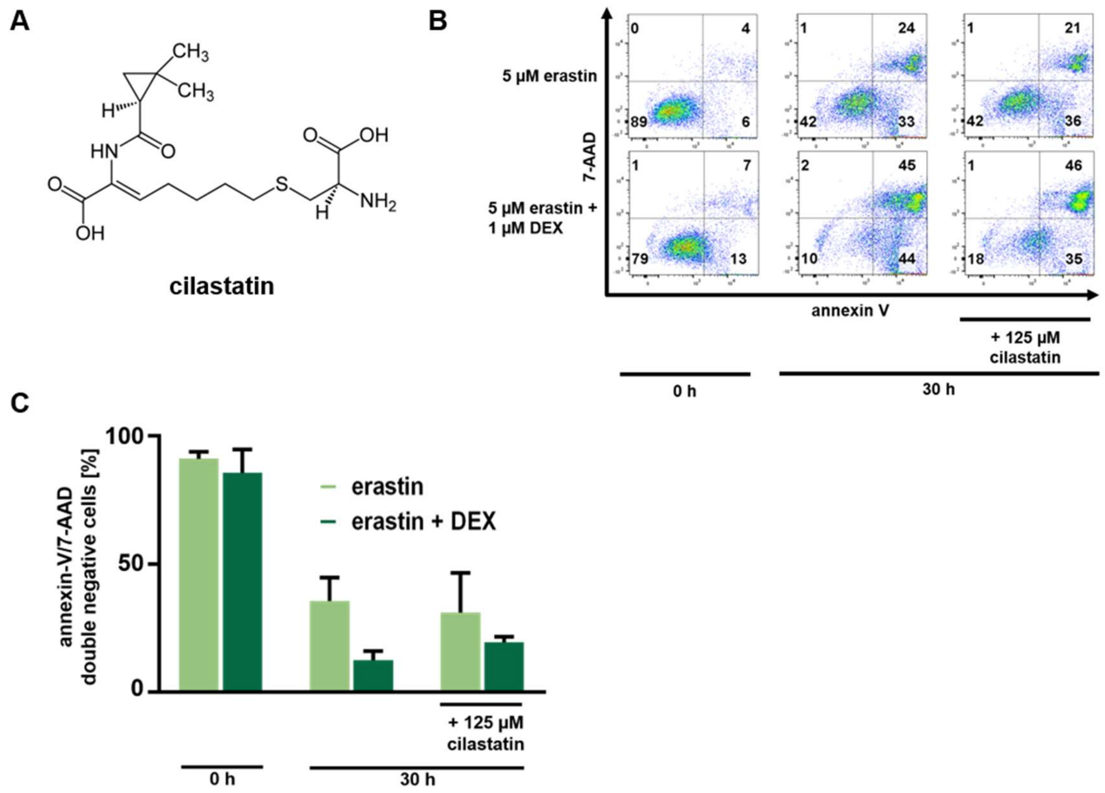

Fig. S9: The dipeptidase-inhibitor cilastatin does not affect erastin-induced ferroptosis in HT1080 cells. (**A**) Structure of the dipeptidase-inhibitor cilastatin. **(B)** HT1080 cells were treated for 30 hours with 5 µM erastin in the prescence or absence of 125 µM cilastatin with ot without pre-treatment of 1 µM dexamethasone for 12 hours. 7-AAD and annexin V were read out by FACS. **(C)** Quantification of data presented in Fig. S5B and repetitions of this experiment. Note that conventional ferroptosis is not affected by co-incubation with cilastatin. The graphs show mean +/- SD.

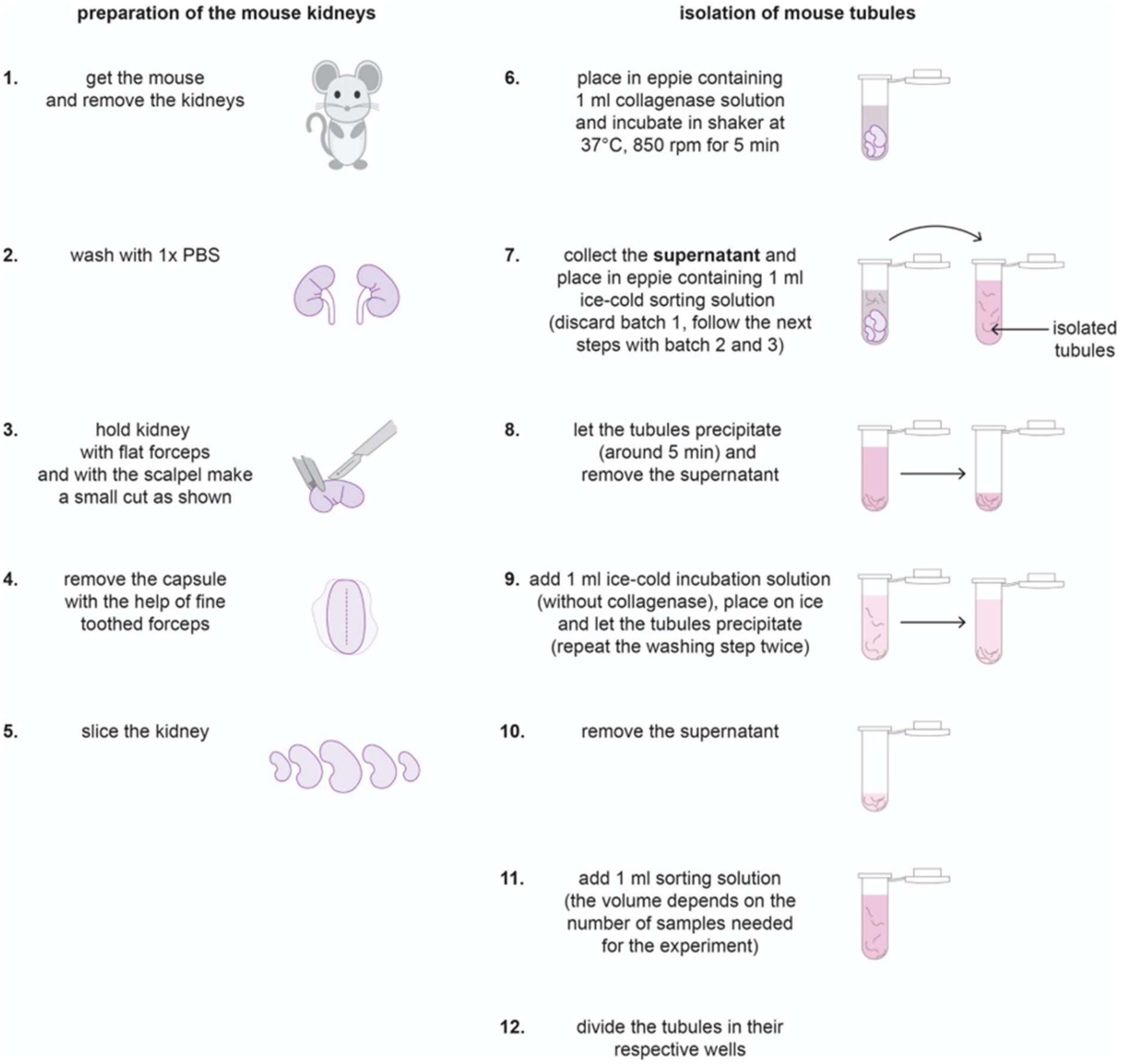

Fig. S10: Protocol for generation of freshly isolated murine kidney tubules. The standard protocol used for generation of the murine kidney tubules as demonstrated in Fig. 5. See methods section for details.

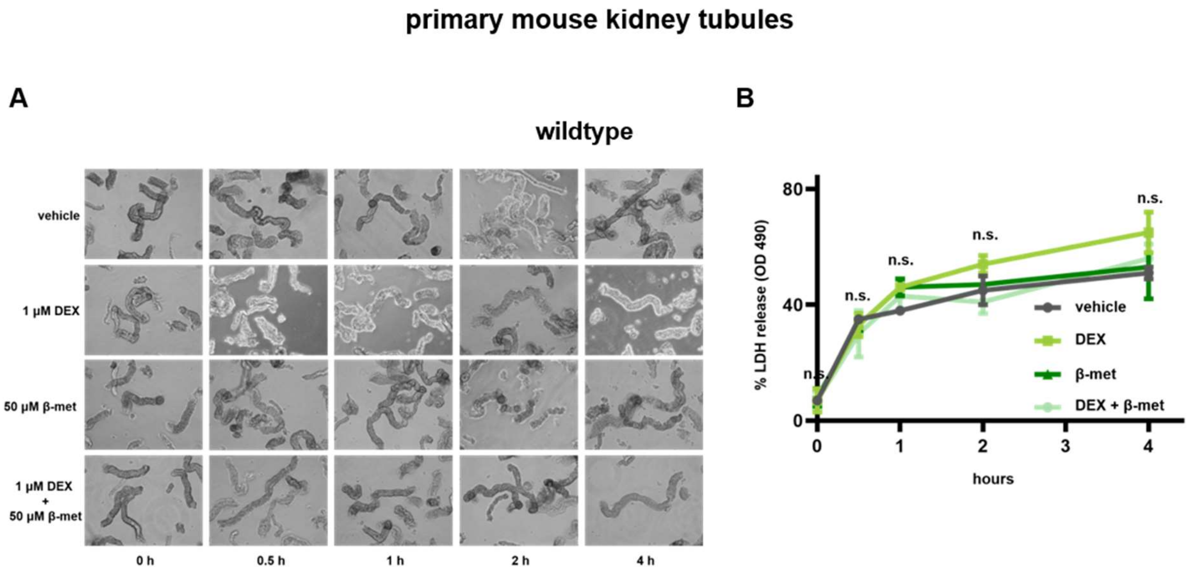

Fig. S11: Acceleration of ferroptosis in freshly isolated renal tubules can be reversed by ß-mercaptoethanol. (**A**) Representative images of freshly isolated murine kidney tubules undergoing spontaneous cell death in the presence of either vehicle or 1 µM dexamethasone (DEX) in the presence or absence of 50 µM ß-mercaptoethanol (ß-met) or ß-mercaptoethanol alone. (**B**) LDH release of respective time points.

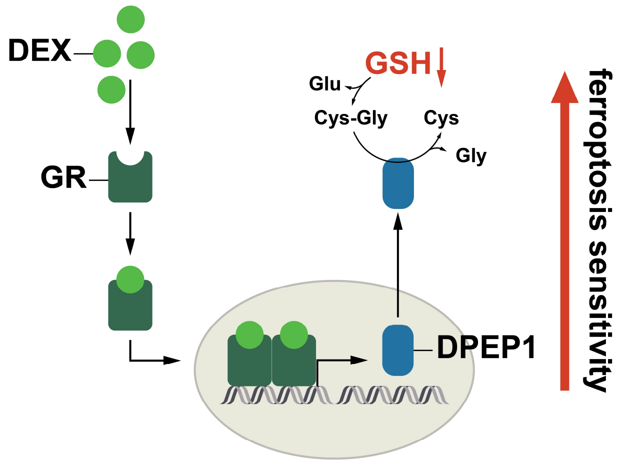

Fig. S12: Model of dexamethasone-licenced sensitization to ferroptosis. Dexamethasone (DEX) triggers dimerization of the glucocorticoid-receptor (GR) that drives the expression of dipeptidase 1 (DPEP1). This enzyme metabolizes cysteinylglycine (Cys-Gly) and thereby decreases concentrations of glutathione (GSH). A GSH depletion generally results in sensitization to ferroptosis.

Video S1: Dexamethasone increases the number of SYTOX green positive cells upon erastin-treatment. Time lapse of HT1080 cells undergoing erastin-induced ferroptosis. SYTOX green is used to visualize membrane permeability which we interpret as necrosis in a 3D printed two chamber system. Note that the movie has been recorded using a single camera. Still images of this video are depicted in **Fig. 1C**.

Video S2: Dexamethasone increases the number of SYTOX green positive cells upon erastin-treatment. Time lapse of HT1080 cells undergoing erastin-induced ferroptosis: Magnification of **Video S1**.

Data S1: Dexamethasone-induced upregulation of mRNAs in HT1080 cells.

Data S2: Dexamethasone-induced downregulation of mRNAs in HT1080 cells
